# Diaphragm Dysfunction and Rehabilitation Strategy in Patients With Chronic Obstructive Pulmonary Disease

**DOI:** 10.3389/fphys.2022.872277

**Published:** 2022-05-02

**Authors:** Yuanyuan Cao, Peijun Li, Yingqi Wang, Xiaodan Liu, Weibing Wu

**Affiliations:** ^1^ Department of Sports Rehabilitation, Shanghai University of Sport, Shanghai, China; ^2^ School of Rehabilitation Medicine, Shanghai University of Traditional Chinese Medicine, Shanghai, China

**Keywords:** COPD, diaphragm dysfunction, structural change, systemic inflammation, inspiratory muscle training, exercise rehabilitation

## Abstract

Chronic obstructive pulmonary disease (COPD) affects the whole body and causes many extrapulmonary adverse effects, amongst which diaphragm dysfunction is one of the prominent manifestations. Diaphragm dysfunction in patients with COPD is manifested as structural changes, such as diaphragm atrophy, single-fibre dysfunction, sarcomere injury and fibre type transformation, and functional changes such as muscle strength decline, endurance change, diaphragm fatigue, decreased diaphragm mobility, etc. Diaphragm dysfunction directly affects the respiratory efficiency of patients and is one of the important pathological mechanisms leading to progressive exacerbation of COPD and respiratory failure, which is closely related to disease mortality. At present, the possible mechanisms of diaphragm dysfunction in patients with COPD include systemic inflammation, oxidative stress, hyperinflation, chronic hypoxia and malnutrition. However, the specific mechanism of diaphragm dysfunction in COPD is still unclear, which, to some extent, increases the difficulty of treatment and rehabilitation. Therefore, on the basis of the review of changes in the structure and function of COPD diaphragm, the potential mechanism of diaphragm dysfunction in COPD was discussed, the current effective rehabilitation methods were also summarised in this paper. In order to provide direction reference and new ideas for the mechanism research and rehabilitation treatment of diaphragm dysfunction in COPD.

## 1 Introduction

COPD is a common respiratory disease caused by exposure to harmful particles or gases, characterised by persistent respiratory symptoms and restricted airflow (Singh et al., 2019). According to the World Health Organization statistical report, COPD is the third leading cause of death in the world, causing 3.23 million deaths in 2019. The high incidence and mortality of COPD make it the fifth disease aggravating the global economic burden. In addition to damaging the airway, alveoli and pulmonary vessels, COPD also damages the circulatory system, nervous system, motor system and other systems, resulting in remarkable extrapulmonary adverse effects (Vanfleteren et al., 2016). Diaphragm dysfunction is present at all stages of COPD development (Donaldson et al., 2012). The diaphragm is the most important inspiratory muscle and the maximum inspiratory pressure is an independent determinant of survival in patients with severe COPD. Diaphragm dysfunction directly leads to dyspnoea and respiratory failure (Elsawy, 2017). It is also associated with increased risk of hospitalisation and increased disease mortality due to acute exacerbation of COPD (Vilaró et al., 2010). Diaphragm dysfunction in patients with COPD is mainly manifested in structural and functional changes, including negative changes and positive adaptive changes. The mechanism of diaphragm dysfunction may be related to systemic inflammation, oxidative stress, hyperinflation, chronic hypoxia, and malnutrition. These factors interact to affect the diaphragm function. Rehabilitation is an individualised and comprehensive treatment plan that is based on existing rehabilitation methods after the assessment of patients’ physique and disease progression. Pulmonary rehabilitation has been shown to be the most effective treatment strategy for improving health outcomes and exercise tolerance in patients with COPD according to the 2022 Global Initiative on Chronic Obstructive Pulmonary Disease guidelines. Studies have shown that rehabilitation could increase diaphragmatic endurance and strength, reduce airflow resistance and improve dyspnoea in patients with COPD, thereby reducing the incidence of diaphragm dysfunction (Gosselink et al., 2011). Therefore, in the present paper, the structural and functional changes and mechanisms of diaphragm dysfunction in COPD were discussed. The common rehabilitation treatment methods of diaphragm dysfunction in COPD, such as Inspiratory muscle training (IMT), exercise intervention and nutritional support, were also summarised to provide reference and new ideas for the rehabilitation and research direction of diaphragm dysfunction in COPD.

## 2 Manifestations of Diaphragm Dysfunction in COPD

### 2.1 Structural Changes of the Diaphragm

The function of diaphragm depends largely on its physiological characteristics at the structural level. Negative changes and positive adaptive changes exist at the same time in the structural changes of diaphragm in patients with COPD. The balance between negative changes and positive changes is closely related to the functional performance and changes of diaphragm in each stage of the disease, which has a profound impact on diaphragm dysfunction and disease progression.

Amongst the negative structural changes of diaphragm in patients with COPD, diaphragmatic atrophy is an important pathological basis of dysfunction (Ottenheijm et al., 2005). Studies have shown a 30% reduction in myosin heavy chain content in patients with mild to moderate COPD and a 30%–40% reduction in diaphragmatic muscle fibre cross-sectional area in patients with severe COPD (Levine et al., 2013). These findings suggested that diaphragmatic atrophy occurs early in the disease and may be present at all stages of the disease. In addition, reduced myosin content in the diaphragm induced changes in the contractile properties and passive properties of diaphragm single fibres in COPD patients. Levine and colleagues’ study showed a 35% reduction in the force generated by diaphragm fibres in patients with severe COPD, indicating a decrease in the contractile properties of diaphragm single fibres (Levine et al., 2003). Moore and colleagues’ data indicated that diaphragmatic fibres exhibit less passive tension during fibre stretching in patients with mild to moderate COPD than in patients without COPD, suggesting a reduction in the diaphragmatic single fibre elasticity (Moore et al., 2006). Sarcomere is the basic structure and function unit of muscle. Patients with COPD have a deletion of serial sarcomeres (Levine et al., 2013). Sarcomere disruption is more evident in patients with moderate and severe COPD(Orozco-Levi et al., 2001). With chronic hyperinflation, there is a 10%–15% loss of sarcomeres in the diaphragmatic muscle fibres (De Troyer, 1997). The biomechanical defects of diaphragm due to changes in diaphragm shape are the main negative factors for diaphragm dysfunction. Pulmonary hyperinflation and deformation of the thorax cause the diaphragm to drop in position, shorten and flatten the normal dome shape (Salito et al., 2015; Laghi et al., 2021). These changes put the diaphragm at a mechanical disadvantage, resulting in a reduced ability of the diaphragm to generate flow and pressure, increasing respiratory work, and causing diaphragmatic fatigue (Hellebrandová et al., 2016).

In addition to the negative changes, there are some positive adaptive structural changes in the diaphragm of patients with COPD. Due to pulmonary hyperinflation, the length of diaphragm muscle segments is shortened in patients with COPD and the greater the lung volume, the shorter the muscle segments are (Orozco-Levi et al., 1999). Muscle segments shortening may result in a partial reversal of the displacement of the diaphragm’s length-tension curve, increasing its ability to maintain tension (Gea et al., 2013), and enhance endurance. The transformation of diaphragm fibre type is the most remarkable structural and physiological characteristic of diaphragm in patients with COPD. The contraction rate and main metabolic types of muscle fibres determine its antifatigue ability. Studies have shown that the diaphragm type II fibres in patients with COPD are transformed into type I fibres with higher oxidation degree, resulting in an increase in the proportion of type I fibres (Nguyen et al., 2000; Levine et al., 2002). To some extent, this transformation is a positive adaptation of the diaphragm muscle. The peripheral vascular network of type I fibre is richer than that of type II fibre and it contains more myoglobin and mitochondria than that of type II fibre. The increase of capillary density, myoglobin content and mitochondrial density can improve the oxygen supply efficiency of diaphragm. Therefore, it compensatively increases the endurance and fatigue resistance of diaphragm (Levine et al., 2003). To some extent, they help the diaphragm adapt to the high load due to increased airway resistance that lowers airflow. However, beyond the compensatory range could cause a decrease in diaphragm endurance. The experimental data of rats show that type I fibres produced less force than type II fibres (Geiger et al., 2000), possibly leading to decreased diaphragm strength in patients with mild to severe COPD.

Therefore, in summary, although positive adaptive changes in diaphragm structure could be found in patients with COPD, negative structural changes still dominate (Levine et al., 2013). In the early and middle stages of the disease, a balance exists between positive and negative changes (Barreiro and Gea, 2015). However, this balance quickly shifts towards negative outcomes as the disease progresses (Gayan-Ramirez and Decramer, 2013).

### 2.2 Functional Changes in Diaphragm

Diaphragm dysfunction could be classified as partial or total loss of diaphragmatic function and it may involve one or both hemidiaphragms (McCool and Tzelepis, 2012). Diaphragm dysfunction in patients with COPD is mostly unilateral diaphragmatic paralysis, which is usually asymptomatic. However, dyspnoea may occur during exertion and supine. (Hart et al., 2002; Steier et al., 2008). From the specific function, patients with COPD with diaphragm dysfunction is mainly manifested as decreased diaphragm muscle strength, endurance changes, reduced mobility, and diaphragm fatigue.

Diaphragm muscle strength mainly depends on muscle fibre cross-sectional area, muscle fibre type and initial length (Mathur et al., 2014). The cross-sectional area of diaphragm muscle fibre in patients with COPD decreases, the muscle fibre type changes from fast twitch muscle to slow twitch muscle and the pulmonary hyperinflation shortens the length of diaphragm muscle (Finucane et al., 2005), resulting in the decline of diaphragm muscle strength in patients with COPD. Levine and colleagues found that although the type conversion of diaphragm fibres increased the ATP production capacity, the utilisation rate of ATP decreased, the specific force decreased, resulting in the weakening of the pressure-generating ability (Levine et al., 2003). Transdiaphragmatic pressure (Pdi) is a more direct indicator of diaphragm muscle strength. The maximum Pdi in patients with severe COPD is only 60% of that in healthy controls (McCool and Tzelepis, 2012). Increased lung volume and altered chest wall geometry led to diaphragm shortening (Silva et al., 2013). An early pioneering study found that the diaphragm of patients was on average 28% shorter than that of healthy subjects (Rochester and Braun, 1985). The diaphragm is shortened to the suboptimal length, which makes the conversion of diaphragm tension into trans-diaphragm pressure ineffective, so as to reduce the pressure-generating ability. Furthermore, pulmonary hyperinflation shortens the operation length of diaphragm and changes the mechanical linkage between its various parts, damaging the muscle strength of diaphragm (Nair et al., 2019).

Patients with COPD showed fragile positive fitness for diaphragm endurance (Papavramidis et al., 2011). The respiratory controller imposes a shorter duty cycle, and the transformation of diaphragm fibre to type II muscle fibre with higher oxidation degree is the main reason for the increase of endurance (Wijnhoven et al., 2006). However, studies have shown that patients with stable COPD have a lower mean diaphragm tension-time index (which indicates the ability of the inspiratory muscles to maintain sufficient pressure generation over time) than normal subjects (Ceriana et al., 2017), indicating decreased diaphragm endurance in patients with COPD. The enhanced endurance adaptations may only be maintained within a certain range. The reasons for this are the low ATP utilization in the diaphragm and the fact that the load on the diaphragm continues to rise with disease severity. Eventually, diaphragmatic endurance is further impaired due to increased muscle weakness and a series of factors that limit regeneration (Macgowan et al., 2001).

Compared with healthy individuals, patients with COPD had reduced diaphragmatic mobility (Gonçalves et al., 2018). Reduced diaphragmatic mobility has been found to be an important factor in decreased exercise tolerance and increased dyspnoea in patients with COPD(Paulin et al., 2007). The maximum level of diaphragm excursion (DE_max_) can fully predict improvement in exercise tolerance in patients with COPD. Another study found a strong correlation between diaphragmatic mobility and maximal voluntary ventilation (MVV) in COPD patients, suggesting that greater diaphragmatic mobility was associated with better ventilatory capacity (Rocha et al., 2017). Increased airway resistance, pulmonary hyperinflation and air trapping are the main factors affecting diaphragm activity. Rocha et al. found that the decrease of diaphragm mobility in patients with COPD was related to airway obstruction (Rocha et al., 2017). Shiraishi et al. (2021). found that reduced diaphragmatic mobility may be associated with a range of symptoms associated with dynamic pulmonary hyperinflation. However, another study suggested that diaphragm mobility correlated strongly with pulmonary function parameters that quantify air trapping, moderately with airway resistance and weakly with pulmonary hyperinflation (Dos Santos Yamaguti et al., 2008).

**FIGURE 1:**
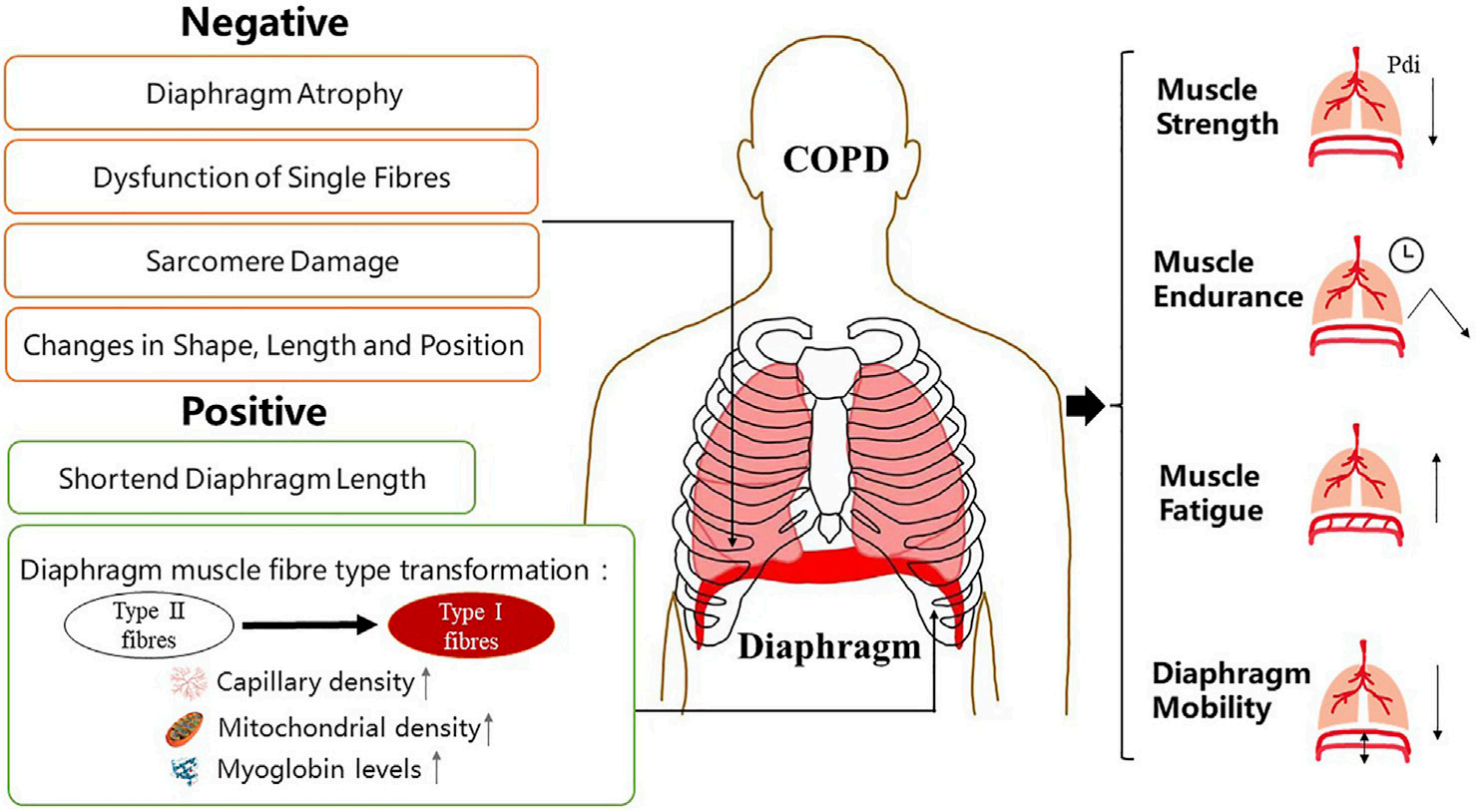
Manifestations of diaphragm dysfunction in COPD. Diaphragm dysfunction in patients with COPD is mainly manifested in structural and functional changes. Changes in diaphragm structure include both negative and positive changes. The function of diaphragm depends largely on its physiological characteristics at the structural level. COPD, chronic obstructive pulmonary disease.

Diaphragmatic fatigue in patients with COPD is characterized by reversible weakening of diaphragmatic contractility and decrease of contractile speed. From an overall point of view, it is the change of muscle output power. Due to hyperinflation and long-term unfavourable mechanical position, the diaphragm is forced to remain in a state of contraction, which is easy to cause the decline of diaphragm compliance and contraction force, leading to diaphragm fatigue (De Troyer, 1997). Additionally, muscle fibre atrophy and damage to the airway and lung parenchymal structure in patients with COPD lead to increased laborious respiratory movement, resulting in reduced fatigue resistance (McKenzie et al., 2009) (Figure 1).

## 3 Mechanisms of Diaphragm Dysfunction in COPD

### 3.1 Systemic Inflammation and Diaphragm Dysfunction in COPD

According to recent studies, COPD is a systemic disease involving chronic low-grade inflammation and altered protein metabolism throughout the body (Mador and Sethi, 2013). Studies have shown that patients with COPD have a systemic inflammatory response, including the activation of inflammatory cells and increased levels of multiple circulating inflammatory factors, such as tumour necrosis factor-*α* (TNF-*α*), interleukin (IL)-1, IL-6, IL-8, and C-reactive protein (Huang et al., 2016). Elevated levels of inflammatory cytokines TNF-*α* and IL-6 were also found in the diaphragm in patients with severe COPD (Barreiro et al., 2011). Studies have shown that the levels of inflammatory factors are negatively correlated with diaphragm quality and muscle strength, suggesting that systemic inflammation is involved in the occurrence and development of diaphragm dysfunction in patients with COPD (Lin et al., 2010). Different studies have found that the negative effects of systemic inflammation on diaphragm dysfunction in COPD may be caused by multiple pathways.

TNF-*α* plays a major role in diaphragmatic catabolism and the levels of TNF-*α* in serum and diaphragm of COPD rats are higher than those of the normal control group, leading to diaphragmatic atrophy. TNF-*α* may directly stimulate total muscle protein, resulting in decreased protein content and loss of muscle-specific protein. It interacts with its receptor to activate caspase-8, thereby initiating the apoptosis pathway. It also activates the nuclear factor-κB (NF-κB) signalling pathway and induces apoptosis or muscle degradation.

NF-κB is an important signalling pathway involved in diaphragmatic mass depletion. Haegens and colleagues’ study reported that NF-κB activation and ubiquitin-proteasome system (UPS) mediated protein degradation are required for pulmonary inflammation-induced diaphragm atrophy (Haegens et al., 2012). Genetic studies have shown that inhibition of NF-κB activity promotes muscle regeneration (Mourkioti et al., 2006). Inflammatory factors may upregulate the expression of E3 ubiquitin ligase atrogin-1/MAFbx and muscle ring finger protein 1 (MURF-1) by influencing the NF-κB signalling pathway, so as to accelerate muscle protein decomposition (Attaix et al., 2005). Activation of NF-κB decreased the expression of MyoD mRNA, disrupted the stability of MyoD protein, and decreased the level of MyoD protein (Shirakawa et al., 2021). It could also inhibit muscle formation by suppressing the expression of growth-stimulating molecules, thus interfering with the regeneration of diaphragm and eventually leading to diaphragm dysfunction (Vogiatzis et al., 2007).

In addition, systemic inflammatory mediators indirectly contribute to muscle atrophy through dysregulation of tissue and organ systems, including GCs via the hypothalamic-pituitary-adrenal (HPA) axis, digestion leading to anorexia-cachexia, and altered liver and adipocyte behaviour, all of which subsequently lead to the development of diaphragmatic atrophy (Webster et al., 2020). The exact relationship between systemic inflammation and the process of diaphragmatic atrophy in COPD patients is currently unknown due to the lack of longitudinal data. The specific mechanisms of inflammatory response involved in diaphragm dysfunction have also not been fully elucidated and should be further investigated.

### 3.2 Oxidative Stress and Diaphragm Dysfunction in COPD

Oxidative stress refers to a state of imbalance between oxidation and antioxidant effects in the body. For the first time, Barreiro and colleagues detected higher levels of oxidative stress in the diaphragm of patients with severe COPD than in control muscles, resulting in increased diaphragmatic muscle depletion and diaphragm dysfunction (Barreiro et al., 2005). Reactive oxygen species (ROS) and reactive nitrogen species (RNS) are the triggering factors of diaphragm dysfunction. A certain amount of reactive oxides has a certain effect on muscle fibre adaptation to exercise or disuse state (Ábrigo et al., 2018). However, high levels of ROS and RNS over a long period of time could lead to proteolysis and potential apoptosis (Ji et al., 2006). Oxidative stress promotes muscle atrophy by activating various proteolytic pathways. UPS is the main pathway for intracellular protein degradation and oxidative stress increases protein degradation in the diaphragm by activating the UPS, resulting in diaphragmatic atrophy (Gomes-Marcondes and Tisdale, 2002; Pomiès et al., 2016). Calprotease is a Ca^2+^-dependent cysteine protease, which could catalyse the restricted hydrolysis of various substrates and plays an important role in the occurrence and development of muscular dystrophy. High ROS levels inhibit sarcoplasmic Ca^2+^-ATPase activity and prevent Ca^2+^ reabsorption into the sarcoplasmic reticulum, thus increasing intracellular free Ca^2+^ concentration and leading to calprotease activation, which promotes muscle degradation (Powers and Jackson, 2008; Whidden et al., 2010). Caspase is closely related to apoptosis and it participates in the apoptosis regulation of muscle cells (Kuwano and Hara, 2000). Oxidative stress can also activate caspase-3 in muscle cells and promote degradation of the complete actin-myosin complex and apoptosis of muscle nucleus cells, resulting in muscle atrophy and loss of muscle nucleus (Du et al., 2004). In addition to the activation of proteolytic enzymes, ROS and RNS modify the diaphragm with protein oxidation, which results in reduced protein activity and makes it more vulnerable to protease degradation. Oxidative damage to muscle proteins prior to characteristic respiratory changes may lead to muscle loss and dysfunction in patients with COPD (Barreiro et al., 2010). Additionally, muscle redox disorder caused by oxidative stress and altered expression of redox-sensitive genes may be responsible for increased susceptibility to diaphragm fatigue (Orozco-Levi, 2003). In conclusion, oxidative stress activates multiple proteolytic pathways, increases protein oxidation and results in altered expression of redox-sensitive genes, leading to diaphragm dysfunction.

### 3.3 Hyperinflation and Diaphragm Dysfunction in COPD

Pulmonary hyperinflation can be due to loss of lung elastic recoil and airway closure at higher volumes, which significantly increases the mechanical load of the diaphragm (Krieger, 2009). The respiratory work of healthy individuals is relatively small and the diaphragm could easily maintain this level of power output. However, the increase of airway resistance places higher demands on diaphragm endurance, which could lead to dyspnoea and reduced exercise tolerance. Hyperinflation results in energy loss and breakdown of muscle proteins, leading to diaphragm atrophy, which makes it even less able to withstand increased stress. Increases in acute and chronic stresses lead to fibre damage and fibrosis of the diaphragm (Scott et al., 2006). During exacerbations of COPD, additional damage to sarcomere may occur when patients are faced with acute diaphragm load, which may further impair diaphragm function (Orozco-Levi et al., 2001), leading to respiratory failure. The increased workload on the diaphragm of patients with severe COPD may also induce oxidative stress and aggravate diaphragm wear by increasing the production of oxidants (Barreiro et al., 2005). Meanwhile, hyperinflation results in changes in diaphragm shape in patients with COPD. A normal diaphragm is located between the thoracoabdominal cavity and it closes the lower thoracic orifice. It is a thin fornix muscle with a flat central part and upward protruding on both sides. The fornix is low on the left and high on the right. By using chest radiographs and spiral CT, Sverzellati et al. found that excessive ventilation resulted in an increase in the radius of curvature of the diaphragm (Sverzellati et al., 2013). Thus, the shape of the diaphragm is nearly flat (Laghi et al., 2021). Normal dome shape plays an important role in diaphragm function. In patients with COPD, diaphragm shape changes and position downshifts away from the optimal mechanical position, resulting in increased respiratory work and diaphragm fatigue (Gayan-Ramirez and Decramer, 2013). In summary, pulmonary hyperinflation increases the dynamic load on the diaphragm, leading to varying degrees of damage and loss of function of the diaphragm cells and subcellular structures. Overloading increases diaphragmatic oxidative stress, leading to biomechanical defects in the diaphragm and ultimately to diaphragmatic dysfunction.

### 3.4 Chronic Hypoxia and Diaphragm Dysfunction in COPD

Patients with COPD suffer from chronic hypoxia with varying degrees of hypoxemia and hypercapnia due to prolonged expiratory flow obstruction, dysfunction of ventilation and retention of sputum. Studies have shown that chronic hypoxia could reduce the aerobic capacity of the diaphragm, resulting in changes in the histological morphology, contractile properties, and metabolism of the diaphragm (Shiota et al., 2004), causing the diaphragm to be unable to cope with the increased load, which may cause diaphragm dysfunction. Histologically, a reduction in muscle fibre cross-sectional area has been observed in the diaphragm in animal models of chronic hypoxia (McMorrow et al., 2011; Gamboa and Andrade, 2012), decreased mitochondrial density and related protein loss in diaphragm muscle (Gamboa and Andrade, 2010). In terms of contractile properties, chronic hypoxia could reduce the rate of diaphragmatic contraction, inhibit the diaphragmatic isotonic properties and power output and reduce the endurance time during repeated isotonic contractions (Machiels et al., 2001). Metabolically, chronic hypoxia reduces diaphragmatic oxygen consumption and ATP production and it may increase dependence on fatty acid oxidation. Activation of hypoxia-inducible factor (HIF), activation of atrophy signal transduction and increased proteolysis due to chronic hypoxia are the main causes of diaphragm dysfunction (Lewis and O'Halloran, 2016). Hif-1 *α*, the subunit comprising HIF-1, is a major regulator of the adaptive response of cells to hypoxia stress and its increased expression limits muscle regeneration (Semenza, 2010). In addition, studies have shown that chronic hypoxia activates TNF-*α*, induces the formation of ROS, and induces high expression of myostatin. These factors can exacerbate systemic inflammation, oxidative stress, and inhibition of myogenesis, respectively (Remels et al., 2007; Gayan-Ramirez and Decramer, 2013), thereby aggravating the damage to diaphragm function. Archiza and colleagues found that female diaphragms were more prone to fatigue under acute hypoxia, which may be related to gender differences in diaphragm metabolism, such as fibre type composition, systolic properties, substrate utilisation and blood perfusion (Archiza et al., 2021). A notable detail that chronic hypoxia could lead to diaphragm dysfunction in patients with COPD, which in turn leads to further hypoxia in a vicious cycle (Bradford, 2004).

### 3.5 Malnutrition and Diaphragm Dysfunction in COPD

Patients with COPD often suffer from malnutrition due to inadequate energy and nutrient intake, gastrointestinal digestive and absorption dysfunction, increased energy expenditure and enhanced catabolism (Holst et al., 2019; Kaluźniak-Szymanowska et al., 2021). According to statistics, 25%–40% of patients with COPD are malnourished (Cano et al., 2004; Vermeeren et al., 2006). Malnutrition causes COPD patients to gradually develop a negative nitrogen balance. The massive consumption of fat and protein causes muscle atrophy and reduced muscle mass. The structural and functional changes of diaphragm caused by malnutrition may evolve into diaphragm dysfunction. At the structural level, malnutrition could directly lead to increased muscle consumption (Xie et al., 2021). Malnutrition is associated with increased diaphragmatic diastolic ratio, decreased muscle weight, decreased fibre size, decreased percentage of type II fibre, and consumption of energy-rich compounds (Orozco-Levi, 2003). At the functional level, malnutrition causes an imbalance in the energy supply and demand of the diaphragm, along with damage to the respiratory system and the body’s defense system. Malnutrition may lead to a reduction in the mass of the diaphragm, which reduces the power to maintain normal ventilation and decreases muscle strength and endurance (Sahebjami and Sathianpitayakul, 2000). In general, malnutrition mainly affects the activity of glycolytic enzymes but it does not impair the function of oxidative pathways. A notable detail that although malnutrition may be associated with diaphragm dysfunction, it is also associated with the severity of malnutrition and its effect is clinically relevant only in some patients. Hamnegard studied diaphragm strength in two groups of 10 patients with severe COPD and found that the levels of malnutrition did not cause diaphragm weakness (Hamnegard et al., 2002), possibly because these changes are related to changes in diaphragm structure without affecting the overall changes in diaphragm function. Structural damage does not necessarily lead to loss of function.

The mechanisms of diaphragm dysfunction in patients with COPD are correlated. For example, oxidative stress could serve as a signal for the expression of inflammatory mediators and inflammation could regulate ROS production and thus oxidative stress levels (Thoma and Lightfoot, 2018). The increased load caused by hyperinflation could induce oxidative stress and aggravate diaphragm wear (Barreiro et al., 2005). Inflammatory factors have been shown to be involved in the development of malnutrition through the fusion of multiple mechanisms (Laviano et al., 2003). Hyperinflation plays an important role in chronic hypoxia and hypercapnia (Zysman et al., 2021). Such interactions may further complicate the condition of diaphragmatic dysfunction. But it also means that partial improvement in each factor has the potential to improve the overall situation. Well-organized rehabilitation has been proven to have a positive effect on diaphragmatic dysfunction in COPD by eliminating adverse triggers and improving functional performance of the diaphragm.

## 4 Rehabilitation Treatment of Diaphragm Dysfunction in COPD

To date, the clinical attention paid to the prevention and treatment of diaphragm dysfunction in patients with COPD is not sufficient. Certain clinical interventions could even aggravate diaphragm dysfunction. For instance, long-term mechanical ventilation could further aggravate diaphragm dysfunction and even makes it difficult to get off the ventilator (Marchioni et al., 2019). IMT, exercise intervention and nutritional support are the main rehabilitation treatment that have been proven to be effective in improving diaphragm function. They are expected to improve diaphragm strength and endurance, enhance the exercise tolerance of patient and improve symptoms such as dyspnoea, overall, improve health-related quality of life (HRQL).

### 4.1 Inspiratory Muscle Training

IMT applies a load to the diaphragm and accessory inspiratory muscles to increase their strength and endurance. It has been proven to improve inspiratory muscle strength, relieve dyspnoea, and improve exercise capacity in COPD patients (Beckerman et al., 2005; Hill et al., 2006; Magadle et al., 2007). The American Thoracic Society/European Respiratory Society (ATS/ERS) guidelines for pulmonary rehabilitation recommend IMT as an effective adjunct to pulmonary rehabilitation. Breathing techniques and neuromuscular electrical stimulation can reinforce and complement the effects of diaphragmatic training. Strengthening of the intercostal muscle synergistically improves diaphragm function and increases the functional reserve of the inspiratory muscles.

#### 4.1.1 Diaphragm Training

Diaphragm training is performed via devices that impose resistive or threshold loads, including inspiratory resistive training and threshold pressure training. Inspiratory resistive training is a method of continuous resistance breathing on the resistive load device at a normal breathing rate. The amount of inspiratory resistance during training depends on the amount of air the patient is breathing. Studies have shown that inspiratory resistive training could combat diaphragm fatigue, increase diaphragm strength and endurance and relieve dyspnoea (Madariaga et al., 2007). In threshold pressure training, the threshold inspiratory muscle trainer is used to set the given domain pressure and the patient’s respiratory work could only reach a certain threshold to open the channel in the trainer and make air flow. Studies have shown that threshold pressure training could enhance the muscle strength and endurance of the diaphragm to relieve dyspnoea and improve lung function (Sturdy et al., 2003; Lee et al., 2021). The threshold pressure training has better effect than inspiratory resistive training (Wu et al., 2017). Notably, recent studies have shown that long-term diaphragm training with high resistance load could lead to diaphragmatic injury, especially in patients with advanced COPD(Bertoni et al., 2019). During diaphragmatic training, patients are usually unable to maintain a high constant load training for a long time due to factors, such as anaerobic respiration, lactic acid accumulation and inadequate perfusion adjustment (Louvaris et al., 2021). In view of this situation, low load should be selected for diaphragm training, which could usually be set to 15–50% of the maximum suction negative pressure, to achieve a relatively safe and efficient improvement of diaphragm function.

Breathing techniques include diaphragmatic breathing and pursed-lips breathing, which can be utilised as compensatory therapy for patients who are unable to exercise (Mendes et al., 2019). Diaphragmatic breathing minimises the respiratory demand of the disease, improving breathing patterns and ventilation efficiency without causing respiratory dyspnoea (Fernandes et al., 2011).Diaphragmatic breathing enables the diaphragm to be fully exercised, thus increasing muscle strength and endurance. Pursed-lips breathing could prevent premature closure of small airways, improve gas exchange and relieve hypoxia symptoms of patients. Studies have shown that pursed-lips breathing could effectively reduce the minute ventilation and respiratory rate during exercise in patients with COPD (Mayer et al., 2018). Pursed-lips breathing can reduce dynamic hyperinflation (de Araujo et al., 2015), thereby reducing the diaphragm load. It could also improve patients’ exercise tolerance (Cabral et al., 2015). Studies have shown that diaphragmatic breathing combined with pursed-lips breathing could increase tidal volume, improve chest wall volume by shortening breathing cycle and relieve diaphragm fatigue by reducing breathing rate (Mendes et al., 2019). Due to the simplicity of implementation and the low incidence of serious adverse effects during training, breathing techniques are suitable for patients with COPD at all stages.

Neuromuscular electrical stimulation trains muscle strength by stimulating the diaphragm and related nerves with a controllable current (Vieira et al., 2014). Studies have shown that neuromuscular electrical stimulation could promote muscle protein synthesis, increase muscle mass and improve diaphragm muscle strength and fatigue resistance (Maddocks et al., 2016), especially for patients with moderate and severe COPD with limited exercise capacity. However, because it is passive training, neuromuscular electrical stimulation should be combined with active exercise to achieve enhanced rehabilitation effect as an alternative to routine rehabilitation exercise.

#### 4.1.2 Intercostal Muscle Training

The contraction of intercostal muscles causes the ribs to move outward and upward, assisting the diaphragm in the coordination of inspiratory completion (Rohrbach et al., 2003). Training the intercostal muscles could increase their strength, endurance and mobility, thereby improving their ability to resist high-load exercise and reducing the load on the diaphragm. Intercostal muscle training mostly adopts exercise that promote upper body activities, such as chest expansion, rotation, swimming and various ball games (Zhou and Sun, 2021). Intercostal muscle training has high compliance requirements for patients and it is suitable for patients with mild to moderate COPD.

### 4.2 Exercise Intervention

Exercise is the core content of pulmonary rehabilitation treatment of COPD. Personalised strength and endurance training plan is the cornerstone of pulmonary rehabilitation. It could effectively improve the diaphragm dysfunction of patients with COPD. Studies have shown that exercise could improve local muscle inflammation and even systemic inflammation in patients with COPD by significantly reducing the levels of inflammatory factors, such as TNF-*α*, in muscle and increasing the levels of anti-inflammatory factors, such as IL-10 (Toledo-Arruda et al., 2017). Exercise also improves oxidative stress in patients with COPD by increasing antioxidant capacity (Vieira Ramos et al., 2018), reducing the activity of oxidase and improving mitochondrial function (Zhang et al., 2021). Long-term exercise could correct chronic hypoxia by improving the oxygen utilisation efficiency of the body. Nutritional status could be improved by increasing appetite and regulating intestinal flora (Donati Zeppa et al., 2021). Thus, exercise could improve diaphragm dysfunction by reducing adverse factors that lead to negative changes in diaphragmatic structure and function as a whole. In addition to the positive effects of direct action on the diaphragm, exercise interventions can train the supporting muscles of the abdomen, back and limbs. These muscles play a better supporting and supportive role during respiration, reducing the fatigue of the diaphragm and thus improving its function.

The constituent elements of the exercise prescription of the training plan include the form, intensity, frequency, and duration of exercise. Aerobic and resistance exercise are the most common forms of exercise used in the rehabilitation of COPD patients. Aerobic exercise is a periodic and dynamic activity involving the major muscle groups of the whole body. It is the basis of the standard training of pulmonary rehabilitation. Lower-extremity endurance training, such as cycling or walking, is the most commonly used aerobic exercise. Studies have shown that aerobic exercise enhances diaphragm endurance and the muscle strength of major muscle groups and relieve diaphragm fatigue (Wada et al., 2016; Burtch et al., 2017). Resistance exercise refers to the active movement of muscles to overcome external resistance, usually with the help of dumbbells, elastic bands and other impedance training equipment (Hoff et al., 2007). Spruit and colleagues reported that resistance exercise significantly improves respiratory muscle strength, exercise capacity and HRQL in patients (Spruit et al., 2002). Panton et al. found that resistance exercise can be a useful supplement to aerobic exercise, effectively improving muscle weakness and atrophy (Panton et al., 2004). The addition of resistance exercise improved functional outcomes in COPD patients currently participating in an aerobic exercise programme (Skumlien et al., 2007). In addition to the positive effect of direct action on the diaphragm, exercise could train the auxiliary muscles in the abdomen, back and limbs for these muscles to play a better auxiliary and supporting role in the breathing process, relieve diaphragm fatigue, and improve diaphragm dysfunction.

With regard to exercise intensity, studies have shown that patients with stable COPD have better clinical outcomes with high-intensity training than with low-intensity training. Higher intensity exercise significantly improves respiratory muscle strength (Gimenez et al., 2000), reduces minute ventilation (Ve), and reduces dyspnoea (Vallet et al., 1997). High intensity here refers to the exercise close to the individual’s peak level, which is operationally defined as reaching at least 60%–85% of the peak working speed during the incremental maximum exercise test, rather than training at high absolute work levels. Similarly, low intensity refers to 20%–40% of the peak working speed during the incremental maximum exercise test (Norton et al., 2010). A single bout of strenuous exercise may increase the level of oxidative stress in the diaphragm in patients with COPD, leading to diaphragm fatigue (Itoh et al., 2004). In addition to being based on objective data, exercise intensity can be developed by the degree of respiratory distress perceived by the patient during exercise. The recommended intensity for resistance training consists of performing one to four sets of 40%–50% resistance of one repetition maximum (RM) for 10 to 15 repetitions ≥2 days per week (Garvey et al., 2016).

Regarding exercise frequency, it is recommended that aerobic exercise is 3–5 times a week and resistance exercise is at least 2 times a week (Spruit et al., 2013). In terms of duration, a 6–12 weeks training program is recommended (Bolton et al., 2013). Some studies have shown that longer-term programs may provide more lasting benefits (du Moulin et al., 2009; Beauchamp et al., 2013).

Noninvasive positive-pressure ventilation (NPPV) includes continuous positive airway pressure, pressure support and proportional assist ventilation (PAV). NPPV is particularly suitable for COPD patients with hypercapnia and mild to moderate respiratory failure (Lightowler et al., 2003; Köhnlein et al., 2014), and can be an important adjunct to exercise training in COPD patients. Hawkins et al. found that compared to spontaneous breathing, the use of PAV in conjunction with exercise training significantly improved exercise capacity and reduced exercise fatigue (Hawkins et al., 2002). Garrod and colleagues report that the use of NPPV as an adjunct to exercise training in COPD patients can help improve exercise tolerance and health status (Garrod et al., 2000). Non-invasive ventilation can reduce hyperinflation (Dennis et al., 2021).The main objective of NPPV in patients with severe COPD is to improve gas exchange and to provide adequate rest for the respiratory muscles after periods of fatigue (García et al., 2011). NPPV assists the diaphragm in respiratory movements, which helps to relieve the burden on the diaphragm, reduce respiratory work and promote the recovery of diaphragmatic dysfunction (Duiverman et al., 2016). However, as it is passive ventilation, the effect of training the diaphragm to contract on its own is lost to a certain extent. It is recommended as an adjunct to exercise training and is not recommended for long-term use.

### 4.3 Nutritional Support

As mentioned above, chronic malnutrition is an important factor in diaphragm dysfunction, and even respiratory failure. Studies have shown that appropriate nutritional support can significantly improve calorie and protein intake, enhance inspiratory muscle function, improve exercise capacity and alleviate dyspnoea symptoms (Weekes et al., 2009; Collins et al., 2013). Nutritional support can improve diaphragm dysfunction in patients with COPD by regulating inflammation, improving oxidative stress and regulating carbon dioxide production (Broekhuizen et al., 2005; Marín-Hinojosa et al., 2021).The main forms of nutritional support include dietary strategy and oral nutritional supplement (ONS).

The dietary strategy is to adjust the daily dietary structure of patients, and provide personalized food fortification, food snacks and dietary suggestions according to the age, illness and nutritional status of patients. Patients with COPD are often complicated with protein-energy malnutrition, so the diet should be supplied with sufficient calories (Akner and Cederholm, 2001). The respiratory quotient (RQ) of carbohydrates is high, which will produce more CO_2_ after oxidation in the body, causing or aggravating CO_2_ retention, aggravating dyspnoea and the burden of diaphragm, so the diet of COPD patients should reduce the energy supply ratio of carbohydrates (Anker et al., 2006). The carbohydrate intake of patients with stable COPD should account for 50%–60% of the total energy, while the carbohydrate intake of patients with acute exacerbation should account for 35%–50%, but the whole day carbohydrate should not be less than 150 g. The RQ of fat is very low. The proportion of fat intake can be appropriately increased. The fat supply of COPD patients in stable stage can account for 20%–30% of the total energy. Patients with COPD suffer from hyperproteinysis, which is also an important factor in diaphragm atrophy. Therefore, a high protein diet should be provided to promote anabolism. Protein supply can be calculated as 1.2–1.5 g/kg body weight per day, accounting for 15%–20% of total energy (Schols et al., 2014). Moreover, adequate vitamins, minerals, and trace elements should also be supplied in the diet.

In order to increase oral nutritional intake, nutritional liquid, semi-solid or powder preparations rich in a variety of macronutrients and micronutrients are added to drinks and foods for oral administration. ONS is often used to supplement intake when food is insufficient to meet the body’s needs. Similarly, ONS can be used as a sole source of nutrition. Sugawara‘s study found that low-intensity exercise therapy with nutritional supplements containing whey peptide can inhibit systemic inflammation, increase muscle strength and improve exercise tolerance in elderly patients with stable COPD (Sugawara et al., 2012). Studies have shown that nutritional supplements such as polyunsaturated fatty acids (PUFAs) and respifor have anti-inflammatory effects and can improve muscle strength and exercise capacity in patients with COPD (Steiner et al., 2003; Broekhuizen et al., 2005). Another study found that L-carnitine could improve exercise tolerance and inspiratory muscle strength in patients with COPD(Borghi-Silva et al., 2006). However, due to the differences in nutritional supplement regimen and effect measurement, more evidence is still needed on the effect of nutritional support therapy in improving diaphragm dysfunction in patients with COPD (Table 1).

**TABLE 1:** Characteristics of included studies.

Study	Group	Sample size	Age, y	GOLD stage	Intervention	Outcome
Beckerman et al. (2005)	EG	21	67.7 ± 3.6	Ⅱ,Ⅲ	IMT:Threshold inspiratory muscle trainer (15 min, 6/wk,48 weeks)	FEV_1_ (pred%); FVC; PImax; 6MWD; SGRQ
	CG	21	66.9 ± 3.3	Ⅱ,Ⅲ	IMT:Fixed load (15 min, 6/wk,48 weeks)	
Madariaga et al. (2007)	EG1	12	62 ± 13.7	Ⅲ, Ⅳ	IMT:Threshold inspiratory muscle trainer (15 min, 2/d, 48 weeks)	PImax; CRQ; PTI; PES
	EG2	11	66 ± 7.2	Ⅲ, Ⅳ	IMT:Resistive load device (15 min, 2/d,48 weeks)	
	CG	10	61.5 ± 8.6	Ⅲ, Ⅳ	usual care	
Hill et al. (2006)	EG	16	69.4 ± 7.2	Ⅱ-Ⅳ	H-IMT: Threshold inspiratory muscle trainer (21 min,3/wk, 8 weeks)	FEV_1_ (pred%); Pdt; PImax; Pthmax; Pthmax/PImax; 6MWD; CRDQ; Sp,O_2_
	CG	17	66.6 ± 9.8	Ⅱ-Ⅳ	S-IMT: Threshold inspiratory muscle trainer (21 min,3/wk, 8 weeks)	
Magadle et al. (2007)	EG	16	65.2 ± 3.4	Ⅱ	IMT: Threshold inspiratory muscle trainer (1 h,3/wk,12 weeks)	FEV_1_ (pred%); PImax; 6MWD; Borg score; SGRQ
	CG	15	66.1 ± 3.2	Ⅱ	S-IMT: Threshold inspiratory muscle trainer (1 h,3/wk,12 weeks)	
de Araujo et al. (2015)	EG	25	64 ± 7	Ⅱ-Ⅳ	PLB (1 year) non-PLB (1 year)	FEV_1_ (pred%); FEV_1_/FVC; 6MWD; TGlittre; DH; mMRC
Maddocks et al. (2016)	EG	25	70 ± 11	Ⅲ, Ⅳ	active NMES (42 sessions, 12 weeks)	FEV_1_ (pred%); FEV_1_/FVC; 6MWD; SGRQ; EQ-5D; CRQ
	CG	27	69 ± 9	Ⅲ, Ⅳ	placebo NMES (42 sessions, 12 weeks)	
Vieira et al. (2014)	EG	11	56.3 ± 11	Ⅲ, Ⅳ	NMES + respiratory physical therapy + stretching exercises (1 h, 5/wk, 8 weeks)	FEV_1_ (pred%); FEV_1_/FVC; 6MWD; T_lim_; TNF-a; PImax; PEmax; Sp,O_2_; Borg score; SGRQ
	CG	9	56.4 ± 13	Ⅲ, Ⅳ	respiratory physical therapy + stretching exercises (8 weeks)	
Wada et al. (2016)	EG	15	61 ± 5.4	Ⅲ, Ⅳ	aerobic exercise+respiratory muscle stretching (2/wk,12 weeks)	FEV_1_ (pred%); FEV_1_/FVC; 6MWD; Borg score; BODE
	CG	15	64 ± 5.6	Ⅲ, Ⅳ	aerobic exercise+upper and lower limb muscle stretching (2/wk,12 weeks)	
Hoff et al. (2007)	EG	6	62.8 ± 1.4	Ⅲ, Ⅳ	maximal strength training (24 sessions, 8-weeks)	FEV_1_ (pred%); FEV_1_/FVC; Peak force; VO_2_peak; Perceived exertion; RER; VE
	CG	6	60.6 ± 3.0	Ⅲ, Ⅳ	normal activity (24 sessions, 8-weeks)	
Skumlien et al. (2007)	EG	40	63 ± 8.0	Ⅲ, Ⅳ	PR: resistance training (4–5/wk) + endurance training (3–4/wk)	SGRQ; 6MWD; maximal oxygen uptake; constant load endurance time
	CG	20	65 ± 7.0	Ⅲ, Ⅳ	non-PR: usual care	
Weekes et al. (2009)	EG	31	68.9 ± 4.0	Ⅲ, Ⅳ	dietary counselling + advice on food fortification (6 m intervention +6 m follow-up)	FEV1 (pred%); FEV1/FVC; BMI; SGRQ; MRC dyspnoea score
	CG	28	69.2 ± 6.0	Ⅲ, Ⅳ	dietary advice leaflet.	
Steiner et al. (2003)	EG	42	66 ± 9.0	Ⅲ, Ⅳ	570 kcal carbohydrate rich supplement (7 weeks)	BMI; CHO take; HGS
	CG	43	68 ± 8.0	Ⅲ, Ⅳ	non-nutritive placebo (7 weeks)	

**Notes**: Data are presented as mean ± SD.

Abbreviations: GOLD, global initiative for obstructive lung disease; EG, experimental group; CG, control group; IMT, intensity inspiratory muscle training; FEV1(pred%), forced expiratory volume in one second; FVC, forced vital capacity; PImax, maximum static inspiratory pressure; 6MWD, 6-min walking distance; SGRQ, St. George’s Respiratory Questionnaire; CRQ, chronic respiratory questionnaire; PTI, pressure–time index; PES, esophageal pressure; H-IMT, high intensity inspiratory muscle; S-IMT, sham inspiratory muscle training; Pthmax: maximum threshold pressure; SpO2: arterial oxygen saturation; training; mMRC, modified Medical Research Council dyspnea scale; PLB, pursed-lips breathing; Non-PLB: test performed without PLB; TGlittre, Glittre-ADL, test; DH, change in inspiratory capacity at baseline; CB, control breathing; EQ-5D, EuroQol 5-dimension; Tlim, time to exercise tolerance; PEmax, maximal expiratory muscle pressure; BODE, Body-mass index, airflow Obstruction, Dyspnea, and Exercise; RER, respiratory exchange ratio; VE, minute ventilation; PR, pulmonary rehabilitation; BMI, body mass index; CHO, carbohydrate; HGS, isometric handgrip strength.

## 5 Conclusion and Prospect

In summary, COPD combined with diaphragm dysfunction is a common phenomenon that directly affects patients’ respiratory status and quality of life and it is closely related to poor prognosis. In stable patients, diaphragmatic injury and adaptation achieved a special balance (Barreiro and Gea, 2015). However, these positive adaptations are not sufficient to restore normal muscle strength and endurance, resulting in increased susceptibility to fatigue and injury and leading to diaphragm dysfunction. No single factor could not fully explain its occurrence. The mechanism of diaphragmatic muscle dysfunction in patients with COPD is a pathway induced by various pathological factors and mechanisms involved in complex processes. Various factors induce protein breakdown, accelerate cell apoptosis, resulting in the loss of diaphragm muscle fibre, leading to diaphragm dysfunction. Rehabilitation methods, such as IMT, exercise intervention and nutritional support can eliminate the risk factors of diaphragm dysfunction to a certain extent, exercise the diaphragm and respiratory auxiliary muscle group locally and as a whole, improve the overall condition, and have a clear rehabilitation effect on COPD patients with diaphragm dysfunction, which can be used as a reference for clinical treatment of COPD patients with diaphragm dysfunction.

In the future, diaphragm dysfunction in patients with mild to moderate COPD should be the focus of research. In terms of the structure and function of diaphragm dysfunction, studies to determine the causal relationship between the presence and severity of COPD and changes in diaphragm structure and function are still lacking. On the one hand, the specific mechanism of inflammatory response, oxidative stress, chronic hypoxia and other factors playing a role in diaphragm dysfunction has not been fully clarified and each theory is not sufficient to fully explain the mechanism of diaphragm dysfunction. On the other hand, most of the current research is animal experimental research and clinical research and dynamic observation are lacking. In the study of rehabilitation treatment of diaphragm dysfunction, the rehabilitation treatment methods have not been unified and a complete and systematic pulmonary rehabilitation training prescription has not been formed. Large-scale clinical trials could be carried out in the future to explore the most suitable individualised rehabilitation programme for patients from clinical practice and to form standardised and unified operation and evaluation standards. A longitudinal dynamic follow-up study could be conducted to explore the correlation between the severity of diaphragm dysfunction and different stages of disease to provide a prospective basis for the early discovery of diaphragm dysfunction.
